# Arachnomelia syndrome in Simmental cattle is caused by a homozygous 2-bp deletion in the molybdenum cofactor synthesis step 1 gene *(MOCS1)*

**DOI:** 10.1186/1471-2156-12-11

**Published:** 2011-01-21

**Authors:** Johannes Buitkamp, Jördis Semmer, Kay-Uwe Götz

**Affiliations:** 1 Bavarian State Research Center for Agriculture, Institute for Animal Breeding, 85586 Grub, Germany

## Abstract

**Background:**

Arachnomelia syndrome is an autosomal recessive inherited disease in cattle. Affected calves die around birth and show malformations of the skeleton mainly affecting the legs, the spinal column and the skull. A number of arachnomelia syndrome affected Simmental calves were recently detected by a surveillance system of anomalies with a peak of more than 120 recorded cases in the year 2006. The causative mutation was previously mapped to a 9 cM-region on bovine chromosome 23. We herein report the fine-mapping and identification of the gene causing arachnomelia syndrome in Simmental cattle.

**Results:**

By using a dense set of markers, the arachnomelia syndrome linked region could be refined to 1.5 cM harbouring three protein coding genes. Comparative sequencing of these genes revealed a two-bp-deletion in the bovine *MOCS1 *gene resulting in a frame-shift and a premature termination codon. We genotyped affected calves and their ancestors and found that all affected were homozygous for the deletion whereas all carriers were heterozygous. Furthermore, cattle from the same population, but not directly related to known carriers mostly showed the wild type genotype.

**Conclusions:**

*MOCS1 *encodes two proteins that are involved in the first synthesis step of molybdenum cofactor. A non functional sulfite-oxydase, one of the enzymes requiring molybdenum cofactor, leads to a similar pathology in Brown Swiss cattle. In combination the perfect association of the mutation with the phenotype and the obvious disruption of protein translation provide strong evidence for the causality of the *MOCS1 *mutation. Our results are the first example for an oligogenic lethal inherited disease in cattle. Furthermore, they show the potential involvement of sulfite metabolism in aberrant bone development.

## Background

The congenital arachnomelia syndrome (AS, OMIA Phene ID 139, Group 000059) in Holstein Friesian, Red Holstein and Simmental cattle was initially described by Rieck and Schade [1]. The cardinal pathological changes are skeletal malformations of the skull, legs and the spinal column [2]. The facial deformation leads to brachygnathia inferior and concave rounding of the maxilla forming a dent ('pointer-head'). The legs show abnormally thin diaphyses of the long bones and stiffened, hyperextended fetlocks ('spider-legs', dolichostenomelia), leading to frequent fractures of the metacarpus and metatarsus in the course of forced birth assistance. The vertebral formation failure results in kyphosis and scoliosis.

In the 1980s, the syndrome was dispersed in European Brown Swiss cattle by the use of American Brown Swiss sires [3] and in 2005 AS reappeared in Simmental. There were more than 150 confirmed cases in this breed with a peak in 2006 [2]. It could be shown by pedigree analysis that AS is most probably a monogenic recessive inherited disease with an estimated allele frequency of 3% in the current cow population [2]. Subsequently, AS was mapped to a 9 cM-region on bovine chromosome 23 by linkage analyses of families including 89 affected Simmental calves [4]. An indirect gene test was developed and applied to several hundred sires that had a pedigree-based risk to be a carrier of AS (results are publicly available [5]). The availability of the indirect genetic test and the awareness of the disease by the artificial insemination centres and breeders led to a sharp decline in cases after 2007 [4]. AS is the first oligogenic Mendelian inherited disease found in cattle, since it was mapped to a different location (on bovine chromosome 5) in Brown Swiss [6].

In this study, we present the fine mapping and identification of the putative disease causing mutation of AS in Simmental cattle thereby providing insights into the genetic basis of AS and the biology of bone development.

## Results

### Fine mapping of the AS region

**Figure 1:**
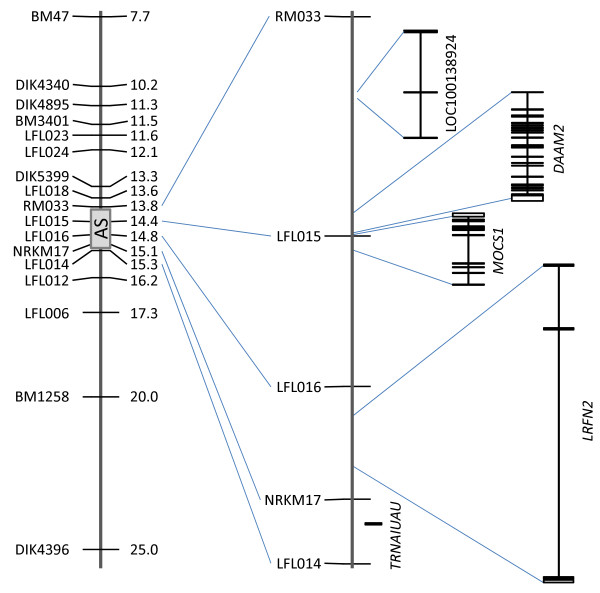
**Partial genetic map of BTA23 and genes located within the arachnomelia syndrome region**. The genetic map of the proximal region of BTA23 is shown at the left side. The region containing the arachnomelia syndrome condition is indicated by a gray rectangle. This region is enlarged in the middle. On the right the location and distribution of exons of annotated genes within the region is shown.

In a previous work we mapped the AS-condition in Simmental cattle to a ~9 cM-region. The corresponding region was covered with additional markers to further narrow down the AS locus. Published [7] as well as newly developed microsatellites were tested within the AS families. Finally, 11 informative additional microsatellites were chosen and the AS families were genotyped (Table 1, Additional file 1). The 86 cases available for fine mapping came from 4 half sib families. Therefore, the paternal and, subsequently, the maternal haplotype could unambiguously be deduced in most cases. 17-marker haplotypes were constructed for all genotyped cases. We analysed the minimal region without recombination and the region with complete homozygosity within the AS-affected animals. Both analyses led to the identical minimal candidate region of AS between the markers RM033 and LFL014 (Figure 1, Table 2). This region is about 0.7 cM or 1.5 Mb in length. These results excluded some prominent candidate genes that are located close to the region in question: *RUNX2 *and *CUL7 *that had been associated with diseases of the skeleton [8,9].

**Table 1:** Microsatellites used for fine mapping of the arachnomelia syndrome locus

Marker name*	**cM**^†^	**App. location (kb)**^‡^	Fragment length range (bp)	Reference	Remarks
*BM47*	13.8	7,765	222-268	This publication; [7]	
*DIK4340*	16.2	10,153	192-204	[7]	
DIK4895	17,9	11,332	174-192	[7]	
BM3401	18.8	11,455	124-146	This publication; [7]	
LFL023	-	11,619	126-194	This publication	
LFL024	-	12,108	172-194	This publication	
*DIK5399*	20.7	13,273	193-201	[7]	
LFL018	-	13,670	215-241	This publication	located within *KCNK17*
RM033	20.7	13,796	146-156	[7]	
LfL015	-	14,354	130-152	This publication	located between *DAAM2 *and *MOCS1*
LFL016	-	14,751	131-187	This publication	
*NRKM17*	21.3	15,050	129-141	[7]	
LFL014	-	15,281	110-134	This publication	
LFL012	-	16,190	118-163	This publication	triple repeat
LFL006	-	17,329	121-201	This publication	located close to *PTK7*
*BM1258*	28.3	19,935	92-106	[7]	
*DIK4396*	39.9	24,934	156-173	[7]	

*Italicized names represent markers that had been used for whole genome linkage analysis [4]; ^†^genetic distance according to [7]; ^‡^according to BTAU4.0

**Table 2:** Haplotypes of arachnomelia syndrome-affected animals of 17 microsatellites of chromosome 23

Marker		arachnomelia syndrome associated haplotypes																														
		
Name*	Chr 23 (Mb) ^†^	1	2	3	4	5	6	7	8	9	10	11	12	13	14	15	16	17	18	19	20	21	22	23	24	25	26	27	28	29	30	31
*BM47*	7.77	232	222	238	238	240	238	254	254	234	268	238	254	268	220	254	238	238	254	232	232	234	240	238	238	254	232	222	238	238	238	234
*DIK4340*	10.16	198	198	204	204	204	204	198	198	198	198	200	198	202	198	198	204	204	198	204	198	200	204	204	198	192	198	204	204	204	204	204
DIK4895	11.33		188	192	192	192	192	190	190	190	190	192	190	174	190	190	192	192	190	192	174	192	192	192	190	190	174	192	192	192	192	192
BM3401	11.46	126	128	140	140	140	140	126	126	126	126	140	126	128	126	126	140	140	126	140	128	140	140	140	126	140	128		140	140	140	140
LFL023	11.62	126	126	126	126	126	126	126	126	126	126	126	126	126	126	126	126	126	126	126	126	126	126	126	126	126	126	126	126	126	126	126
LFL024	12.11	188	178	190	190	190	190	178	178	178	178	190	178	178	178	178	190	190	178	190	184	190	190	190	178	188	184		190	178	190	190
*DIK5399*	13.27		197	197	197	197	197	197	197	197	197	197	197	197	197	197	197	197	197	197	199	197	197	197	197	197	199		197	197	197	197
LFL018	13.67	225	227	227	227	227	227	227	227	227	227	227	227	227	227	227	227	227	227	227	227	227	227	227	227	225	227	227	227	227	227	227
RM033	13.80	148	150	150	150	150	150	150	150	150	150	150	150	150	150	150	150	150	150	150	150	150	150	150	150	150	150	150	150	150	150	150
**LFL015**	**14.35**	**144**	**144**	**144**	**144**	**144**	**144**	**144**	**144**	**144**	**144**	**144**	**144**	**144**	**144**	**144**	**144**	**144**	**144**	**144**	**144**	**144**	**144**	**144**	**144**	**144**	**144**	**144**	**144**	**144**	**144**	**144**
**LFL016**	**14.76**	**133**	**133**	**133**	**133**	**133**	**133**	**133**	**133**	**133**	**133**	**133**	**133**	**133**	**133**	**133**	**133**	**133**	**133**	**133**	**133**	**133**	**133**	**133**	**133**	**133**	**133**	**133**	**133**	**133**	**133**	**133**
***NRKM17***	**15.05**	**135**	**135**	**135**	**135**	**135**	**135**	**135**	**135**	**135**	**135**	**135**	**135**	**135**	**135**	**135**	**135**	**135**	**135**	**135**	**135**	**135**	**135**	**135**	**135**	**135**	**135**	**135**	**135**	**135**	**135**	**135**
LFL014	15.28	130	130	130	130	130	130	130	130	130	130	130	130	130	130	130	130	130	130		130	112	130	130	130	130	130	130	130	130	130	130
LFL012	16.19	160	160	163	163	163	163	160	160	160	160	160	160	160	160	145	163	163	160	142	157	145	163	160	160	160	160	163	163	163	163	163
LFL006	17.33	193	193	193	193	193	193	193	195	193	193	193	193	193	193	193	193	193	193	197	193	191	193	193	193	193	193	193	193	193	173	173
*BM1258*	19.94	96	96	98	98	98	98	96	98	96	96	96	96	96	96	96	102	98	96	100	96	102	98	98	98	96	98	98	98	98	102	102
DIK4396	24.94	162		165	167	167	162	162	165	162	162	165	165	162	162	150	167	173	167	162	162	164	162	165	162	167	165		165	165	167	167

The minimal genetic region is shown in bold; *Italicized names represent markers that had been used for whole genome linkage analysis [4]; †according to BTAU4.0

### Investigating candidate genes within the region carrying the AS mutation

The 1.5 Mb-core candidate region on chromosome 23 harboured one pseudogene, a TAF9 RNA polymerase II, one UAU-encoding tRNA gene, and three protein-coding genes, *DAAM2*, *MOCS1*, and *LRFN2*, that were annotated in BTAU4.0 (Figure 1). Based on their functional annotation none of these genes was a straightforward candidate with a known function in bone or cartilage development. The pseudogene is similar to *TAF2G *encoding an ubiquitously expressed transcription factor that is essential for cell viability [10]. *DAAM2 *encodes the dishevelled associated activator of morphogenesis 2 that is involved in the wnt signalling pathway [11] implying a role in early cell development. Nevertheless, the gene function is not very well understood and there are no data from disease associations or animal models. *MOCS1 *encodes the molybdenum cofactor synthesis 1 protein that is involved in the synthesis of the molybdenum cofactor (Moco). Moco is required by at least three enzymes, sulfite-oxidase, xanthine-dehydrogenase, and aldehyde-oxidase. Known mutations within *MOCS1 *lead to an autosomal recessive disease, the molybdenum cofactor deficiency, with neurological symptoms in humans [12]. The last gene, *LRFN2 *encoding the leucine-rich repeat and fibronectin type III domain-containing protein 2, is known to interact with different receptors in brain tissue and plays a role in t-cell development and hematopoiesis [13]. Nevertheless, the protein seems to interact with the NMDA receptors [14] that, among other functions, are involved in bone development [15,16]. Since there was no convincing candidate, we systematically sequenced the exonic and part of the intergenic, 5' and 3' regions of all three genes. The genomic organization of the bovine *DAAM2 *and *MOCS1 *was derived from the BTAU4.0 annotation, whereas the exons of *LRFN2 *were deduced from human and mouse EST and RNA data, since the first exon was not annotated. In total 22.4 kb were sequenced and twelve mutations, including one deletion, were detected within the three genes by comparative sequencing of one unrelated, one AS-carrier, and two AS-affected animals (Table 3).

**Table 3:** Results from the comparative sequencing of the three positional candidate genes of affected and unaffected cattle

Name	N exons	kb sequenced	N mutations	position and type of mutations		
*DAAM2*	24	13.91	2	c.2017c > g	synonymous	exon 16
				c.2145 + 22c > t	-	intron 16
*MOCS1*	11	4.63	2	c.711t > c	synonymous	exon 6
				c.1224-	frameshift	exon 7
				1225delCA		
*LRNF2*	3	3.86	8	c.-72a > g	-	intron 1
				c.-87a > g	-	intron 1
				c.-116a > g	-	intron 1
				c.216t > c	synonymous	exon 2
				c.525a > g	synonymous	exon 2
				c.622a > c	synonymous	exon 2
				c.927g > a	synonymous	exon 2
				c.1095t > c	synonymous	exon 2

A panel of additional animals was tested for these alleles either by direct sequencing, allele-specific PCR or PCR-RFLP assays. Excluding (either an AS-affected calf being not homozygote or a known carrier being not heterozygous for the mutation and the wt allele) genotypes were found for all mutations, except for the deletion within *MOCS1*.

### Mutation of the bovine *MOCS1 *gene

**Figure 2:**
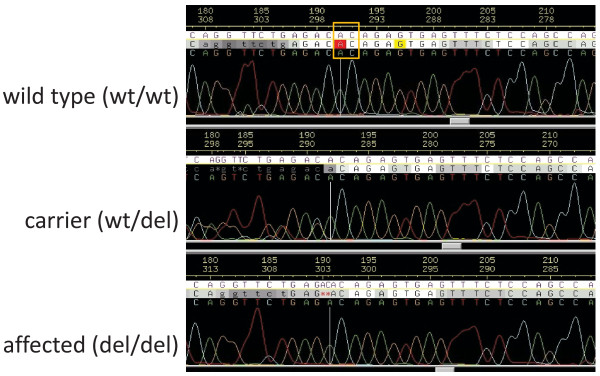
**Sequence tracks encompassing the arachnomelia syndrome-causing mutation within the bovine *MOCS1***. Selected sequence tracks enclosing the c.1224-1225delCA mutation. From top to bottom electropherograms of a wild type (wt/wt), a carrier (wt/del), and an arachnomelia affected calf (del/del) are shown. The CA dinucleotide at postion 192-193 (indicated by the yellow box) of the wild type sequence is deleted in the sequence of the affected calf.

The two bp-CA-deletion (Figure 2) is located at RNA (GI:261490660) position 1224-1225 (numbering starting at the A of the translation start codon) that is located in exon 11 of *MOCS1 *(c.1224-1225delCA). A genotyping system based on allele specific PCR was designed for the mutation and all cases as well as the family members available were genotyped (Table 4). As expected, all affected calves were homozygote for the mutation (del/del), whereas all parents were heterozygote (wt/del). As a control, cattle sampled randomly from the Bavarian Simmental population excluding first-degree relatives of known carriers were genotyped. None of them was homozygote for the c.1224-1225delCA mutation, but about 2.8% were heterozygote. The mutation was not observed in 120 cattle from other breeds (German Gelbvieh, Holstein Friesian, Belgian Blue and Braunvieh). We analysed the pedigrees of the heterozygous Simmental cattle from the random sample and found that, as previously observed for the affected calves, all could be traced back to SEMPER or his sire SENAT, confirming the hypothesis, that SENAT may be the founder and that most of the carriers inherited the mutation via SEMPER [2].

**Table 4:** Genotype frequencies of the *MOCS1 *deletion

	wt/wt	wt/del	del/del
Affected calves^†^	0	0	154
Carriers	2*	219	0
Unrelated Simmental cattle	599	17	0
Cattle from four other breeds	241	0	0
Total	843	236	154

^†^The parents of arachnomelia syndrome-affected calves. *The arachnomelia syndrome signs of the affected calves that are descendents from these two sires are considered to be phenocopies [2].

Finally, 67 sires that were known to be putative carriers of the mutation, either by pedigree analysis or by results from genetic testing [5], were genotyped. These sires include ROMEL and REXON, that produced a large number of daughters. All 67 sires were heterozygous for the deletion. DNA from the suspected founder, SEMPER, was not available, but DNA from one of his sons (SENDER) and brothers (SET) were genotyped. These were homozygous for the wild type allele. This result is in agreement with the finding, that all pedigrees available can be traced back to SEMPER via one of his daughters, but not to one of his sons. Two affected calves, that were considered as phenocopies since there was no connection of their pedigrees with SEMPER or SENAT [2, Table 4] were homozygote for the wild type allele.

**Figure 3:**
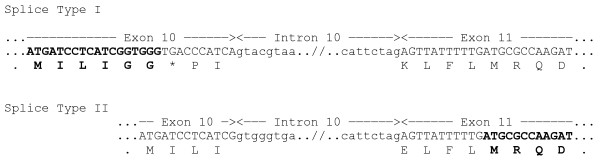
**Alternative splicing of the bovine *MOCS1***. Part of the genomic sequence and the deduced amino acids of exon 10, intron 10 and exon 11 of the bovine MOCS1 is shown. Exon-intron boundaries are indicated by arrows above the nucleotide sequence. The coding sequences of the predicted RNAs are shown with capital letters, intronic sequence with small letters indicating the putative splice sites leading to splice type I and II. Splice type II leads no the no-nonsense transcript (predicted RNA accession number GI:261490660) that is translated into a functional MOCS1B protein. The end of MOCS1A (top) and the beginning of MOCS1B protein sequences (bottom) are shown in bold.

*MOCS1 *is one of the rare genes known to express a polycistronic RNA. It produces two different enzymes (MOCS1A and MOCS1B) from non-overlapping open-reading-frames from a bicistronic transcript [12]. The RNA is known to occur in different splice types in vertebrates including mouse, human, cattle and invertebrates [17]. At least two splice types seem to exist in cattle, encoding either MOCS1A or a functional MOCS1B that is translated as a fusion with an inactive MOCS1A protein (Figure 3).

**Figure 4:**
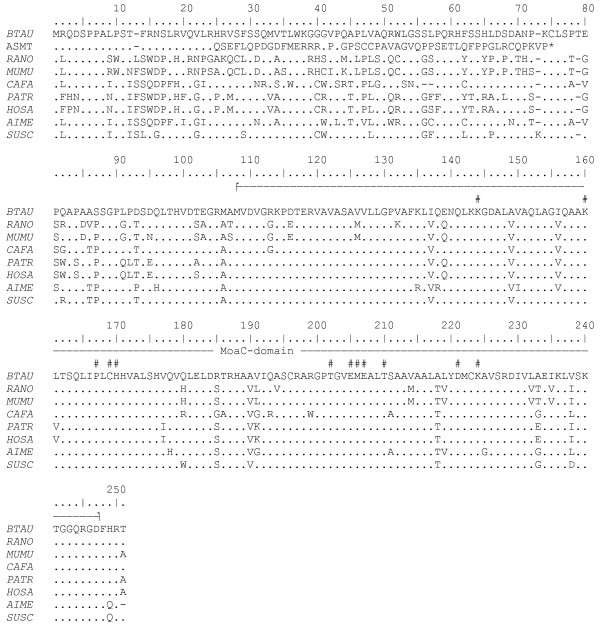
**Comparison of deduced MOCS1B amino acid sequences among cattle, rat, mouse, dog, chimpanzee, human, giant panda and pig and the mutated protein causing the bovine arachnomelia syndrome**. Sequences were derived from GenBank (*BTAU, Bos taurus, gi 261490661; RANO, Rattus norvegicus, gi 149069513; MUMU, Mus musculus, gi 161484628; CAFA, Canis familiaris, gi 73972793; PATR, Pan troglodytes, gi 114607308; HOSA, Homo sapiens, gi 30913216; AIME, Ailuropoda melanoleuca, gi 281353482; SUSC, Sus scrofa, gi 19403921*). The second line shows the predicted MOCS1B amino acid sequence of calves carrying the arachnomelia syndrome mutation (ASMT) The *line *above the sequence alignment indicates the MoaC-domain and *number signs *indicate the highly conserved amino acid residues that are thought to be involved in the biosynthesis of precursor Z [31].

The reference sequence (GI:261490661) of the bovine MOCS1 is 633 amino acids in length and the C-terminus encoding the Moa-C domain is highly conserved within mammals (Figure 4). The 2 bp deletion leads to a predicted frame shift beginning at amino acid position 24 of MOCS1B. The altered protein is 73 amino acids in length and the complete MoaC domain is skipped. Therefore, the altered protein is unlikely to be functional.

## Discussion

The emergence of the bovine AS caused serious concern among European Simmental breeders in 2006, since the disease was transmitted by some top sires that had been extensively used. Therefore, we mapped the AS locus and an indirect gene test was made available in summer 2007 that was an effective tool to control the disease by genotyping sires with conspicuous pedigrees.

To explore the disease mechanism and to avoid the drawbacks of an indirect gene test, the causative mutation should be identified based on the initial mapping data. Additional regional markers were applied and the AS region was finally narrowed down to a 1.5 Mb-region containing five genes. Since none of them could convincingly be associated with the phenotype of AS, three genes were comparatively sequenced. As a result, exactly one obviously functional mutation in *MOCS1 *could be identified. Genotyping of a large panel of affected, obligate carrier and unrelated cattle provided additional evidence, that we identified the genuine AS mutation for Simmental cattle.

*MOCS1 *encodes a bicistronic RNA that is translated into MOCS1A and MOCS1B that are involved in the first step of the synthesis of Moco. They are analogous to the MoaC and MoaA enzymes of *Escherichia coli *and promote the synthesis of precursor Z (or cyclic pyranopterin monophosphate, cPMP) from a 5'-GTP derivate. Mutations of *MOCS1 *that lead to an inability of synthesising functional precursor Z cause Moco-deficiency type A (MIM 252150) in human, an autosomal recessive inherited disease that is mainly characterised by neurological symptoms due to brain dysmorphisms (loss of white matter in the CNS) [18]. Nevertheless, mutations of an other gene leading to a disturbed sulphur metabolism, *CBX*, cause homocystinuria that is associated with different signs including abnormally developed phalanges (arachnodactyly) [19].

Homozygous *MOCS1 *knock-out mice show curly whiskers, die between day 1 and 11 of age and are smaller than wt mice, but do not show any other morphological abnormalities or significant changes in CNS [20]. *MOCS1*^-/- ^mice show the biochemical characteristics of sulfite and xanthine intoxication, e.g. elevated sulfite and xanthine levels and undetectable levels of uric acid in the urine. The main pathological mechanism seems to be mediated by the sulfite oxidase deficiency (SOD), since, probably due to redundancy, known isolated deficiencies of other molybdenum dependent enzymes (aldehyde oxidase and xandthine oxidoreductase) do not result in clinical symptoms in humans [21,22]. The precise mechanisms of this toxicity are not completely understood. In mice, cell death appears to be triggered by elevated sulfite levels [23].

All splice forms of *MOCS1 *are found in a constant ratio in a wide range of tissues, probably reflecting its universal use [24]. In cattle the main predilection site of Moco-deficiency is the developing bone. Even though bone malformations are not common signs of Moco-deficiency in human and mice, rarely microcephaly and a prominent forehead are observed in patients with Moco- [25] and SOD-deficiency [26].

Mice and children with Moco-deficiency are usually born alive. It was postulated that sulfite is ameliorated in affected embryos by maternal clearance and therefore damage seems to be modulated till birth [23]. Most probably this is not the case in cattle due to the non-invasive implantation. Therefore, (bone-)tissue is not protected from damage from elevated sulfite levels.

Finally, the causative role of *MOCS1 *in bovine AS is strongly supported by the recent discovery of a *SUOX *mutation leading to a similar phenotype in Brown Swiss cattle [27]. Therefore, AS is the first known example for an oligogenic inherited disease in cattle caused by mutations within genes of an enzyme and its cofactor. These results provide impetus for investigating the possible role of elevated sulfite levels in aberrant bone development.

Moco can be treated by purified precursor Z in mouse [23]. In cattle it will not be possible to treat Moco in near future, since the critical insult occurs during fetal development and affected calves usually are not viable postnatally. Instead, AS is controlled by excluding carriers of the mutation from breeding. Replacing the indirect by a direct gene test will accelerate the eradication of AS from the Simmental population. Since the direct gene test is not only more efficient but also easier and cheaper than the indirect gene test, it will become feasible to test larger numbers of breeding cows. This is important due to the comparatively high frequency of carriers in the current cow population.

## Conclusions

Our results strongly support the hypothesis that a missing molybdenum cofactor, leading to non functional Moco-dependent enzymes, namely sulfite oxidase, lead to aberrant bone development in bovine AS.

There are 4 lines of evidence for the mutation of *MOCS1 *being causative for AS. 1) AS is an autosomal recessive inherited lethal disease. Therefore, it could be expected that a loss of function mutation is the cause of AS. 2) The genotyping results of additional animals show complete concordance of the deletion genotype with the AS phenotype. 3) Mutations of *MOCS1 *or the dependant *SUOX *can also lead to bone malformations in human. 4) The corresponding mutation of *SUOX *leads to a similar phenotype in Brown Swiss cattle.

## Methods

### Collection and DNA extraction

The collection of AS-affected Simmental calves had been described previously [4]. In short, affected calves were identified in the course of the Bavarian surveillance programme for inherited congenital malformations. Only calves with AS status confirmed by pathological investigation were included in the study. DNA from tissue was prepared using the DNA tissue kit (Qiagen). DNA from blood was prepared using a blood kit (PEQLAB Biotechnology GmbH). Pedigrees were extracted from the joint German and Austrian Simmental pedigree data used for routine genetic evaluation.

For determination of allele frequencies Simmental cattle were collected from the performance testing station reflecting the breeding population of the years 2002 - 2008 when the AS mutation was spread in the population by some prominent sires [2]. Cattle with parents being known carriers were excluded from the analyses. Holstein Friesian and Braunvieh cattle were male calves bought from local breeders in Bavaria.

### Microsatellite genotyping

DNA from pedigree members were genotyped with 17 microsatellites covering the region 13-40 cM on chromosome 23 (Table 1, Additional File 1). These were selected from the map published by Ihara [7] or developed based on the bovine RefSeq assembly BTAU4.0 (Table 1). One primer of each pair was fluorescently labelled and microsatellites were amplified in 10 μl-reactions and separated on an ABI Prism 310 Genetic Analyzer (Applied Biosystems) as described [4]. Fragment sizes were measured and allele tables were generated using the GENEMAPPER (V. 3.0, Applied Biosystems) software.

### Comparative sequencing

Exonic as well as part of the intergenic, 5'- and 3'-regions of three genes were amplified in 10 μl reaction volume using 2 μl of genomic DNA, 0.05 μM of each primer (Supplement 1), 50 μM of each dNTP, 0.5 units of HotStar-taq polymerase (Qiagen, Hilden, Germany), and reaction buffer containing 1.5 mM MgCl_2_. Cycling-conditions on a Biometra T gradient 96-well thermocycler were: 15 min at 95°C, [0.5 min at 95°C, 1 min at 60°C, 0.5 min at 72°C]35 × and 10 min, 60°C final extension. Before direct sequencing PCR-reactions were purified using the QIAquick PCR Purification Kit. Sequencing was performed using BigDye V 3.0 terminator cycle sequencing kit (Applied Biosystems, Foster City, CA). Sequencing analysis was run on an ABI PRISM^® ^310 genetic analyser. Single nucleotide variation was analysed using PolyPhred [28-30].

### Allele specific PCR genotyping system

Primers (5'-cctgacatgaacaggggaac-3', 5'- ccaggtgggaactgaagtgt-3', 5'-tggagaaactcactctgtgtctc-3', 5'- gtgttcaggttctgagacagagt-3') were designed in order to amplify the two alleles of the 2-bp deletion in an tetra-ARMS reaction. 1.5 pmol of each primer were used to amplify 30 ng genomic DNA in a 10 μl PCR reaction with 0.5 units of HotStar-taq polymerase (Qiagen, Hilden, Germany) under standard conditions. Cycling-conditions on a Biometra T gradient 96-well thermocycler were: 15 min at 95°C, [0.5 min at 95°C, 40 sec at 60°C, 1 min at 72°C]29×. The fragments were separated on a 2% agarose gel in TAE-buffer at 150 V for 40 min. The PCR reaction resulted in fragments of 208 bp and 142 bp in length for the wt and AS allele, respectively.

## Authors' contributions

JB drafted the manuscript, designed the mapping and sequencing strategy and analyzed the data. Genotyping and sequencing was done by JS for wet-lab portions.

K-UG participated in study design and coordination and critically revised the manuscript. All authors read and approved the final manuscript.

## Supplementary Material

Additional file 1 **BTA23 microsatellite primer sequences**. Name and sequences of up and down primers used to amplify the BTA23 microsatellites used for fine mapping of arachnomelia syndrome. Click here for file

Additional file 2 **Primers for comparative sequencing of candidate genes**. Name, location and sequences of primers used for comparative sequencing of candidate genes. Click here for file
