# Supplementary material for: Arachnomelia syndrome in Simmental cattle is caused by a homozygous 2-bp deletion in the molybdenum cofactor synthesis step 1 gene (MOCS1)
Source: BMC Genet. 2011 Jan 21;12:11. doi: 10.1186/1471-2156-12-11 (PMC3034695; doi:10.1186/1471-2156-12-11)
Supplement: Additional file 1 — BTA23 microsatellite primer sequences. Name and sequences of up and down primers used to amplify the BTA23 microsatellites used for fine mapping of arachnomelia syndrome. [file 1471-2156-12-11-S1.DOC]

| Marker name | Primer 1 (5´-3´) | Primer 2 (5´-3´) |
| --- | --- | --- |
| BM47 | ACAGGAAGGAGAAGGGGAAG | CCTTAAGAAGGGCCGAAAGT |
| DIK4340 | TCTATTGGGAGCCCAGAGTC | AACAGTTGAATCTCCTCTTTTCT |
| DIK4895 | GGGAAAGCCCAAAAGGTTTA | ACTTTCTGTCCAGGGCTGAG |
| BM3401 | GCTTCTTGCTCCACCAATTC | AGGGAACCTCAAGCTGATGA |
| LFL023 | agccatctgggaacctctg | gcttccctgggagtgaaaat |
| LFL024 | tgcaaacagaacagcagaaa | gggagggggatatcattcac |
| DIK5399 | TCACCAGATGCCTTTCCTTC | TCAGGTGGTAGCATTTGACG |
| LFL018 | ggctgggatcagagtggtta | ggtggacacggagaagaaga |
| RM033 | TTGCTCCCTGAGCTCATTCT | GTTTTCTTGTGGGAGGTGGA |
| LfL015 | GCTTCCCTCAGCTTCGTTTA | AAAGTCATCCATGGCACACA |
| LFL016 | ACCAATGACAAGCCTGATCC | CTGCTCTGCTGGAGCCTTTA |
| NRKM17 | CTGGTTAGTGATAGGTAGCT | CTTAGACAGGGAGACAGGA |
| LFL014 | tctgaaagggaatggtctcc | cctgcctgctctgtctgtta |
| LFL012 | cctctgctgaacccctgag | cctggtaggctgcagtcc |
| LFL006 | gtggacaagaaggtcccaga | ccaaggtgctctctgctctc |
| BM1258 | GTATGTATTTTTCCCACCCTGC | GAGTCAGACATGACTGAGCCTG |
| DIK4396 | CAGACTCTTGTCCATTTCCTGA | TTGCCTGGAGAATCCTATCG |
