# Supplementary material for: Arachnomelia syndrome in Simmental cattle is caused by a homozygous 2-bp deletion in the molybdenum cofactor synthesis step 1 gene (MOCS1)
Source: BMC Genet. 2011 Jan 21;12:11. doi: 10.1186/1471-2156-12-11 (PMC3034695; doi:10.1186/1471-2156-12-11)
Supplement: Additional file 2 — Primers for comparative sequencing of candidate genes. Name, location and sequences of primers used for comparative sequencing of candidate genes. [file 1471-2156-12-11-S2.DOC]

| Gene | Used for sequencing of region | Sequence 5'-3' |
| --- | --- | --- |
| *LRFN2* | exon 3 | gtctgggacgtggaagaaaa |
| *LRFN2* | exon 3 | caacgagctcatggacttca |
| *LRFN2* | exon 3 | gcagctcctcctttctctga |
| *LRFN2* | exon 3 | ctgtgtatccggctctgaca |
| *LRFN2* | exon 2 | gcacagtttgtccctggaat |
| *LRFN2* | exon 2 | ggaggacttggacctctcct |
| *LRFN2* | exon 2 | agtttctgcaggtcggtgaa |
| *LRFN2* | exon 2 | aagctgttccaaggcgataa |
| *LRFN2* | exon 1 | ctcaaacggaactcgctctc |
| *LRFN2* | exon 1 | ctccctcctagctccatgc |
| *DAAM2* | exon 1 | acccagatcacgatgaggac |
| *DAAM2* | exon 1 | taagaggatcccggggaaat |
| *DAAM2* | exon 2 | gacagaagcttgagggcttg |
| *DAAM2* | exon 2 | ccaggctaaagatccagcag |
| *DAAM2* | exon 3 | cctgaggttcggatgtttgt |
| *DAAM2* | exon 4 | tcctcccttctcttgagcac |
| *DAAM2* | exon 5 | tgcaaaatgcagaccgaata |
| *DAAM2* | exon 5 | tcgagggtcactttccaaac |
| *DAAM2* | exon 6 | tttggaaagtgaccctcgac |
| *DAAM2* | exon 6 | cagggggcaagacatgatac |
| *DAAM2* | exon 7 | tgaagcgcatgactaacagg |
| *DAAM2* | exon 7 | ggatcactgccctaggttga |
| *DAAM2* | exon 8 | gggacaggcatgagatccta |
| *DAAM2* | exon 9 | gggatcaagttctgcgtgtt |
| *DAAM2* | exon 10 | atgctcagggtaccaacagg |
| *DAAM2* | exon 10 | gcgtcttgtgcgtgatttta |
| *DAAM2* | exon 11 | gctgcacagattggagatca |
| *DAAM2* | exon 13 | gcaggctgtggatctatggt |
| *DAAM2* | exon 14 | cctctgccatttgtccactt |
| *DAAM2* | exon 14 | cccaggccagaaaaagaata |
| *DAAM2* | exon 15 | aaacacacacgtcagccttg |
| *DAAM2* | exon 16 | gcctcaagcccctaaaaaga |
| *DAAM2* | exon 17 | tcccttggagcagatgaaac |
| *DAAM2* | exon 18 | agaagggtgccagttgctta |
| *DAAM2* | exon 19 | caggcaggtgtggagaagat |
| *DAAM2* | exon 20 | tgcctgcagtggtaactgac |
| *DAAM2* | exon 21 | ccaagagacgcacaatcaga |
| *DAAM2* | exon 22 | cgcacatctgtcgaatagga |
| *DAAM2* | exon 23 | atggggatctcccctctcta |
| *DAAM2* | exon 24 | agcaccaagtcgcgttaagt |
| *DAAM2* | exon 25 | gtgatgagagcgcgtgatta |
| *DAAM2* | exon 26 | gcctctcattgcaggaaatc |
| *DAAM2* | exon 10 | ggagcttgctgaggagattg |
| *DAAM2* | exon 11 | cggcaacagcaagactgata |
| *DAAM2* | exon 13 | gagctgggatcttcaggtca |
| *DAAM2* | exon 5 | ccacggtctaggtaagagtgcat |
| *DAAM2* | exon 16 | gaccagagtactcacatcgctg |
| *DAAM2* | 5-prime | aggactaggccttcccagag |
| *DAAM2* | 5-prime | caaccaccctctgcagtaca |
| *DAAM2* | 3-prime | cggagaagttttcgacaagg |
| *DAAM2* | 3-prime | ctcacctgggaaggagacac |
| *DAAM2* | 3-prime | atgaaccttgctggagttgg |
| *DAAM2* | 3-prime | aaagaagggagaacggaagc |
| *DAAM2* | 3-prime | cgtatattgttccagggctca |
| *DAAM2* | 3-prime | gggaacccaaaggtgaattt |
| *DAAM2* | 3-prime | ggtggcagtacccatttcct |
| *DAAM2* | 3-prime | gtggggaaggatgatgactg |
| *MOCS1* | exon 1 | aatcctgtgtcagggaatgc |
| *MOCS1* | exon 1 | ggcggtcgatctagagaaca |
| *MOCS1* | exon 2 | gtgtgtgcaggtgtcagagg |
| *MOCS1* | exon 3 | ggtggcaggactctttgaac |
| *MOCS1* | exon 4 | gggagcacttggtgtttgat |
| *MOCS1* | exon 4 | caggctgtttccagagaagg |
| *MOCS1* | exon 5 | gggacattcaccatgaatcc |
| *MOCS1* | exon 8 | ttcaccatcactcccgtgta |
| *MOCS1* | exon 9 | gcctctcattgcaggaaatc |
| *MOCS1* | exon 10 | aagcagcagtaaaggctgga |
| *MOCS1* | exon 11 | acctcctggtgaggaaacct |
| *MOCS1* | exon 11 | gagagggatcagctgactgg |
| *MOCS1* | exon 6 | tccatgagaagcctccaatc |
| *MOCS1* | exon 7 | cccactgagcgtgtaggaat |
| *MOCS1* | exon 2 | tccttgcttgtcaagtggggt |
| *MOCS1* | exon 7 | ttctcatggagagcgttggtg |
| *MOCS1* | exon 8 | attggaggcgaggttcgcag |
| *MOCS1* | exon 11 | ttctcactgtgctcttggccc |
| *MOCS1* | exon 1 | tgtgccgcgcggctttctg |
| *MOCS1* | exon 2 | ctgaaccccacttgacaagc |
| *MOCS1* | exon 8 | ggactccctgcttcactgac |
| *MOCS1* | exon 8 | gacccaagcaatgatggaac |
| *MOCS1* | exon 7 | ctgacccagaggcagagaac |
| *MOCS1* | exon 7 | ctccctccgtgacatcagac |
| *MOCS1* | exon 11 | cctgacatgaacaggggaac |
| *MOCS1* | exon 11 | gagagggatcagctgactgg |
